# Photosensitized Thermoplastic Nano-Photocatalysts Active in the Visible Light Range for Potential Applications Inside Extraterrestrial Facilities

**DOI:** 10.3390/nano12060996

**Published:** 2022-03-17

**Authors:** Lidia Mezzina, Angelo Nicosia, Fabiana Vento, Guido De Guidi, Placido Giuseppe Mineo

**Affiliations:** 1 Department of Chemical Sciences and INSTM UdR of Catania, University of Catania, V.le A. Doria 6, I-95125 Catania, Italy; lidia.mezzina@phd.unict.it (L.M.); angelo.nicosia@unict.it (A.N.); fabiana.vento@phd.unict.it (F.V.); guido.deguidi@unict.it (G.D.G.); 2 Institute for Chemical and Physical Processes, National Research Council (IPCF-CNR), Viale F. Stagno d’Alcontres 37, I-98158 Messina, Italy; 3 Institute of Polymers, Composites and Biomaterials, National Research Council (IPCB-CNR), Via P. Gaifami 18, I-95126 Catania, Italy

**Keywords:** photocatalysis, copolyacrylates, visible light radiation, porphyrins, depollution, space facilities

## Abstract

Among different depollution methods, photocatalysis activated by solar light is promising for terrestrial outdoor applications. However, its use in underground structures and/or microgravity environments (e.g., extraterrestrial structures) is forbidden. In these cases, there are issues related to the energy emitted from the indoor lighting system because it is not high enough to promote the photocatalytic mechanism. Moreover, microgravity does not allow the recovery of the photocatalytic slurry from the depolluted solution. In this work, the synthesis of a filmable nanocomposite based on semiconductor nanoparticles supported by photosensitized copolyacrylates was performed through a bulk in situ radical copolymerization involving a photosensitizer macromonomer. The macromonomer and the nanocomposites were characterized through UV-Vis, fluorescence and NMR spectroscopies, gel permeation chromatography and thermogravimetric analysis. The photocatalytic activity of the sensitized nanocomposites was studied through photodegradation tests of common dyes and recalcitrant xenobiotic pollutants, employing UV-Vis and visible range (λ > 390 nm) light radiations. The sensitized nanocomposite photocatalytic performances increased about two times that of the unsensitized nanocomposite and that of visible range light radiation alone (>390 nm). The experimental data have shown that these new systems, applied as thin films, have the potential for use in indoor deep underground and extraterrestrial structures.

## 1. Introduction

In the last few decades, xenobiotic pollutants that are particularly refractory to biodegradation have been released into the environment, especially in water [1,2], causing severe repercussions on the environment and human health [3]. Additional risks arise from emerging anthropogenic pollutants, such as pesticides, fertilizers, personal care products, and pharmaceuticals, as well as their metabolites or their degradation products [4], whose impact on humans and the environment is not fully clear yet [5].

Recently, increasing interests have been focused on indoor pollution emitted from furniture and human activities within closed environments, representing a health risk, especially for those organisms subjected to long-term exposure. This occurs both in conventional (homes, offices, malls, etc.) and unconventional environments, such as terrestrial deep underground laboratories (DULs) or extraterrestrial and low Earth orbit (LEO) structures. As an example, indoor pollutants in the International Space Station (ISS) [6,7,8,9,10,11] represent a non-negligible problem due to the scarceness of primary resources such as water and air. Thus, their purification and recycling are crucial aspects [12,13,14] and must be conducted through energy-saving methods.

Such aspects become fundamental, especially considering the future space missions that have the intention for humans to settle on the Moon (Artemis project by NASA) and, subsequently, to reach the surface of Mars, which would oblige the crew in long travel within a closed vehicle.

In particular, advanced oxidation processes (AOPs) allow the degradation of pollutants through the production of highly reactive species, such as hydroxyl radicals [15], capable of oxidizing (reduction potential E = +2.80 V) and degrading many organic pollutants in aqueous solutions, attacking C-H and C-C bonds in a non-selective way [16].

Among the AOPs, photocatalysis has already shown its efficiency in on-Earth applications and appears very promising for the abatement of organic pollutants [17,18], exploiting solar radiation as a renewable and infinite energy source to produce reactive oxygen species [19,20]. Titanium dioxide nanoparticles (TiO_2_ NPs) are the most diffused semiconductor-based photocatalysts, because they are cheap, easy to find, non-toxic [21] and efficient against xenobiotic pollutants. Nevertheless, there are two main issues surrounding their applications, regarding both their efficiency and practicality.

The first issue relates to their large bandgap (3.2 eV), making them inoperable for indoor applications because the energy range emitted from the indoor illuminations systems would not be sufficient to promote the photocatalytic mechanism [22]. The resolution of this issue is also necessary for applications in sites that cannot take advantage of solar lighting (both underground constructions [23,24] and extraterrestrial/low Earth orbit structures [25] or, in the future, lunar shelters). An archetype of these kinds of structures is represented by the ISS, where the old illumination system has been recently replaced with low-energy light sources, which do not emit in the light region <390 nm [26,27].

To overtake this issue and extend the photo-response towards the visible light region, different approaches have been proposed [28,29,30,31,32]. Some examples are represented by the doping of the semiconductors with metal or non-metal atoms [33,34,35] or the coupling with other semiconductors [36,37,38,39]. A promising alternative that was recently suggested is represented by organic dyes sensitization [40].

In particular, various semiconductors have been photosensitized with numerous dyes [41], such as xanthene derivatives (Rose bengal, Eosin, etc.) [42], transition metal complexes [43], phthalocyanines [44] and porphyrinoids [45,46]. In particular, porphyrinoids are well-known for their high molar extinction coefficients in the visible range and their light-harvesting ability [47,48]. Their versatility is exploited in multi-faceted applications [48], such as for the preparation of sensors [49,50,51,52,53] or theranostic agents [54,55,56,57].

The photocatalyst sensitization could be performed through different methods: physical or chemical adsorption, covalent linkage, sol-gel method or by mixing the two components [58,59,60]. Photocatalysts based on porphyrin-sensitized TiO_2_ take advantage of light radiation within the visible range to efficiently perform the photodegradation of common model dyes (such as methylene blue) [58] and real xenobiotic pollutants such as antibiotics [61], but also the reduction of CO_2_ to CO [62]. This represents a promising approach, especially for indoor application, thanks to the possibility to tune the working wavelength of the sensitized photocatalyst.

The other fundamental issue regarding TiO_2_ (also photosensitized) arises from their use as a suspended nanopowder in wastewater treatment, leading to the need to separate the semiconductor NPs from the depolluted water [28,63]. For unconventional living environments, these kinds of operations appear to be difficult for different reasons [64,65], especially in the case of structures kept in microgravity [66]. To avoid these issues, polymers could be exploited as support for photocatalyst nanoparticles. Among the several methods proposed to obtain polymer-supported photocatalysts [67,68,69,70], the in situ polymerization approach has recently shown its capability to enhance the photodegradation efficiency of TiO_2_ NPs, thanks to a local waveguide effect which takes places among the different moieties of the nanocomposite itself [71,72]. Polymethyl methacrylate (PMMA) might be considered as the ideal support since it is transparent to UV-Vis radiation, chemically and mechanically stable and it is usable in thin film form [73].

Based on what was discussed before, the development of thermoplastic co-polymeric nanocomposites, having optimized photocatalytic activity in the visible light range, seems a potential solution to provide energy-saving auxiliary systems, lengthening the time of use of the recycling and purification systems already employed in both on-Earth and out-of-Earth indoor environments.

To this aim, here we focused on the design of sensitized photocatalytic PMMA-based nanocomposites containing TiO_2_ as the active photocatalyst, through a synthetic approach specifically designed to ensure the intimate contact between photosensitizer and photocatalyst nanoparticles. A physical blend between the polymer matrix, the photosensitizer and the photocatalyst could lead to different issues, i.e., the migration inside the polymer matrix of both the photocatalyst and photosensitizer, resulting in aggregation phenomena, decrease in photocatalytic performances and the leak of photocatalytic materials.

A suitable acrylic macromonomer, named MAP, containing the photosensitizing unit as a side-group, was synthesized by Steglich reaction between the methacrylic acid (MAA) and the 5-(4-aminophenyl)-10,15,20-triphenyl porphyrin (PNH_2_). Co-polyacrylates-based nanocomposites were synthesized through a bulk in situ radical co-polymerization, involving methyl methacrylate (MMA) and MAP as co-monomers and TiO_2_ nanoparticles as photocatalyst. The obtained products and their precursors were characterized through MALDI-TOF mass spectrometry, NMR, UV-Vis and fluorescence spectroscopies, gel permeation chromatography and thermogravimetric analysis.

The photocatalytic performances of all the sensitized nanosystems were investigated by using them in the form of thin films within tubular glass reactors. Preliminary photocatalytic tests were performed using methylene blue (MB) and rhodamine B (RB) as model probes [74], and common herbicides and drugs (paraquat and acetaminophen, respectively) were used to verify the effectiveness against xenobiotic recalcitrant pollutants found in wastewater (and also foreseeable in future extraterrestrial settlements). The efficiency of the in situ obtained nanosystem was also compared with that of a suitably prepared physical blend between an MMA-MAP copolymer and TiO_2_ nanoparticles.

Finally, the photocatalysts’ performances were verified both under UV-Vis radiation and with UV-shielded light, confirming the boosted activity of the photosensitized nanosystems.

This study demonstrated that this approach increases up to two times the photocatalytic performance of the sensitized nanocomposite compared to that of the un-sensitized one, when irradiated by UV-Vis light. To our knowledge, this is the first case reported about a thermoplastic photocatalyst suitable for working at λ > 390 nm.

## 2. Materials and Methods

Methyl methacrylate (MMA) and AIBN were purchased from Fluka.

5-(4-aminophenyl)-10,15,20-triphenyl porphyrin (PNH_2_) was purchased from Frontier Scientific and Aeroxide^®^ P25 from Acros Organics (21 nm primary particle size (TEM), 35–65 m^2^/g (BET)).

1-methyl-5-(p-toluoyl)-pyrrole-2-acetic acid sodium salt dihydrate, *N*,*N*′-dicyclohexylcarbodiimide (DCC), *N*,*N*-dimethylpyridin-4-amine (DMAP), methacrylic acid (MAA), tetrahydrofuran (THF), n-hexane, ethanol (EtOH), water (LC-MS grade), anhydrous dichlorometane (CH_2_Cl_2_), chloroform (CHCl_3_), deuterated chloroform (CDCl_3_) and triethylamine (TEA) were purchased from Sigma Aldrich, St. Louis, MO, USA.

### 2.1. Syntheses

#### 2.1.1. Synthesis of the 5-(4-Methacrylamide)-10,15,20 Triphenyl Porphyrin (MAP)

**Scheme 1:**
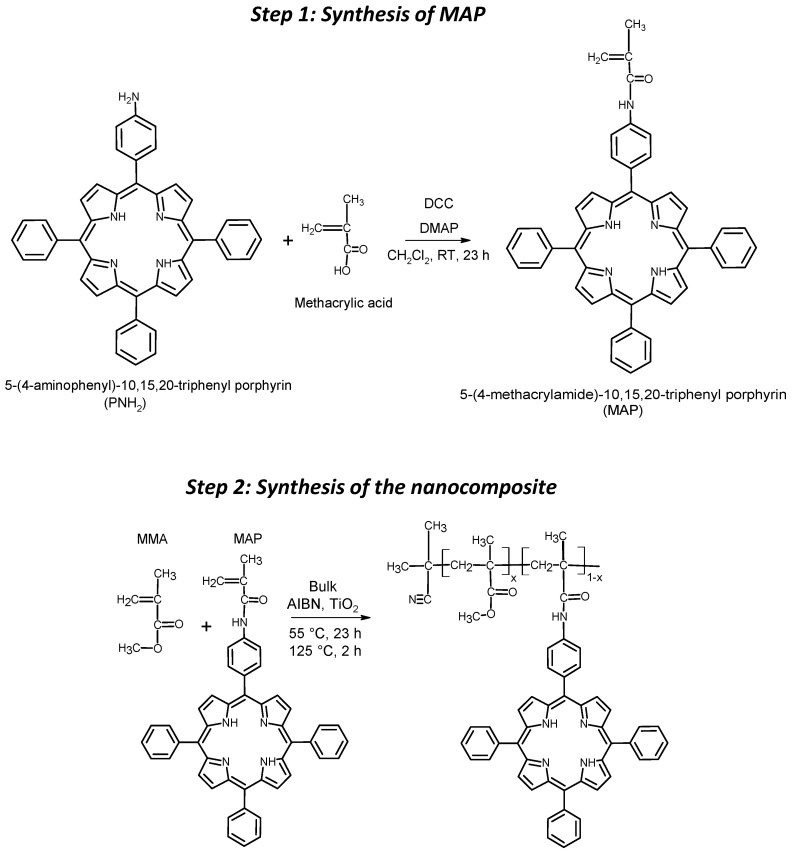
Simplified synthesis schemes of the MAP monomer (step 1) and of the photosensitized nanocomposites (step 2).

The macromonomer was synthesized through Steglich amidation reaction between 5-(4-aminophenyl)-10,15,20-triphenyl porphyrin (PNH_2_) and methacrylic acid (Scheme 1, Step 1).

Briefly, 100 mg (160 μmol) of PNH_2_, 41 mg (475 μmol) of methacrylic acid, 150 mg (700 μmol) of DCC and 43.5 mg (360 μmol) were solubilized, respectively, in 5 mL of anhydrous CH_2_Cl_2_ each. Firstly, methacrylic acid and DCC were mixed, working in an anhydrous nitrogen atmosphere, and stirred in an ice bath. After 15 min, DMAP and PNH_2_ were added to the mixture. The reaction was carried out under a nitrogen atmosphere and at room temperature for 23 h. Then, the mixture was dried with a nitrogen stream and the reaction products were separated via chromatography using a silica-packed column with a mixture of CHCl_3_/EtOH/TEA (99.4:0.5:0.1) as eluent. The macromonomer MAP was the second colored product eluted from the column (yield of about 55%).

^1^H-NMR (CDCl_3_, 500 MHz): 8.85 ppm (m, 8H, C-H pyrrole protons in **2,3,7,8,12,13,17,18** positions); 8.21 ppm (m, 8H, C-H phenyl protons, **a**, **a’**); 7.96 ppm (m, 2H, C-H phenyl protons, **b’**); 7.85 ppm (s, 1H, N-H amide proton, **c**); 7.75 ppm (m, 9H, C-H phenyl protons, **b**); 5.97 ppm (s, 1H, C-H vinyl proton, **d**); 5.60 ppm (s, 1H, C-H vinyl proton, **e**); 2.21 ppm (s, 3H, C-H methyl protons, **f**); −2.78 ppm (s, 2H, N-H pyrrole protons, **g**). Calculated mass C_48_H_35_N_5_O 697.82 Da; experimental, MALDI-TOF: 698.9 Da (detected as MH^+^ species).

#### 2.1.2. Synthesis of the Nanocomposites and the BlendP-TiO_2_

To obtain the photoactive nanocomposites, a bulk in situ radical copolymerization was carried out (Scheme 1, Step 2). Briefly, MAP (in variable quantities depending on the desired loading weight %), TiO_2_, AIBN and MMA were put in a vial and homogenized in an ultrasonic bath for 30 min to guarantee the adsorption of the two co-monomers inside the pores of TiO_2_ NPs. Then, the vial was put inside a silicone oil bath at 55 °C for 23 h and at 125 °C for 2 h. Next, the obtained product was recovered with THF (5 mL) and precipitated in n-hexane. Finally, the nanocomposite was dried in a vacuum oven at 50 °C for 24 h. The same procedure was adopted to produce also the photosensitizer-free nanocomposites, by a bulk in situ polymerization involving methyl methacrylate and TiO_2_, obtaining the nanocomposite (NC).

Through the same reaction steps, a copolymer made of MMA and MAP (copolyMMA-MAP) was produced and used to prepare the BlendP-TiO_2_. Briefly, 15.18 mg of the copolymer was solubilized in THF and mixed with a THF suspension of TiO_2_ NPs (0.5 mL, 1.5 mg/mL). The mixture was homogenized in an ultrasonic bath for 15 min and subsequently deposited via solvent casting onto the inner walls of the glass test tube (see the photocatalytic experiments section).

### 2.2. Photocatalytic Experiments

**Figure 1:**
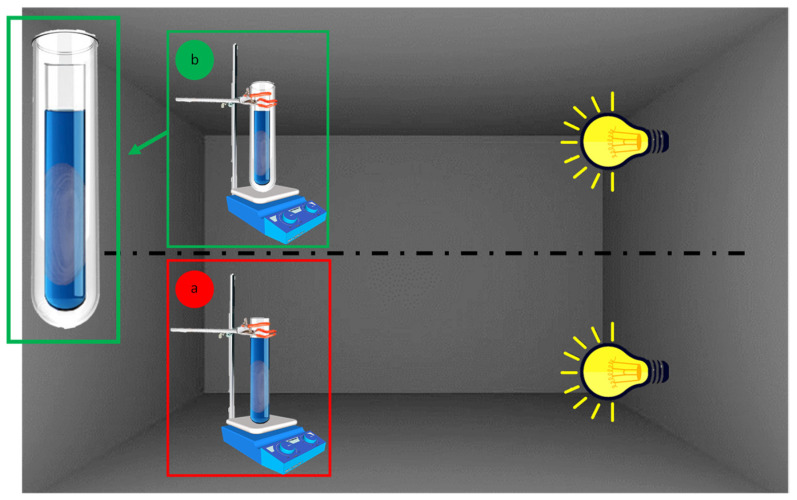
Experimental setup for photocatalytic tests without (**a**) or with (**b**) filter (cut-off ≤390 nm).

Photocatalytic experiments were carried out inside a homemade apparatus (Figure 1a) composed of a 150 mL glass test tube (from here called reactor) kept at a fixed distance of 25 cm from a solar lamp (OSRAM ultra vitalux, Wattage 300 W, OSRAM Opto Semiconductors GmbH, Regensburg, Germany). To avoid any influence of light radiation from both natural and artificial sources, this apparatus was installed into a blacked-out fume hood.

To carry out photocatalytic tests excluding UV radiations, an appropriate chemical filter (500 μM 1-methyl-5-(p-toluoyl)-pyrrole-2-acetic acid sodium salt dihydrate aqueous solution) capable of shielding radiations lower than about 400 nm was used. Then, the reactor was plunged inside the aqueous solution containing the chemical UV filter (Figure 1b).

For each nanocomposite, about 15 mg of sample were dissolved in 1.5 mL of THF and deposited on the inner wall of the glass reactor, taking care of homogeneously covering only 180° of the circumference (the covered surface area was about 55 cm^2^), and left to dry at 50 °C for 12 h.

For each experiment, 100 mL of an aqueous solution of the chosen pollutant was prepared at a specific concentration (absorption spectra are reported in Figure S1).

To avoid any overestimation of the photodegradation performance due to pollutant physisorption phenomena, the systems were subjected to a dark storage time before the lamp was turned on (t_0_, C_0_).

To quantify the magnitude of the photo-oxidation due to the presence of the photocatalytic film, the variation of the concentration (C/C_0_) during the light exposure time was monitored through UV-Vis spectroscopy, analyzing solution aliquots. Considering that the used pollutants follow pseudo-first-order degradation kinetics, the linear-fit slope of the ln(C/C_0_) vs. time plot represents the photodegradation kinetic constant (k) of the pollutant under study. Finally, the k was normalized with respect to the weight of TiO_2_ loaded inside the nanocomposite (norm.k).

### 2.3. Instruments

MALDI-TOF mass spectra were acquired with a Voyager DE (PerSeptive Biosystem, Perkin Elmer, Waltham, MA, USA), detecting the positive ions in linear mode by using the delay extraction device (25 kV applied after 2600 ns, a potential gradient of 454 V mm^−1^ and a wire voltage of 25 V) [75,76]. The samples were prepared using trans-2-[3-(4-tert-butylphenyl)-2-methyl-2-propenylidene] malononitrile (DCTB) as a matrix. The mass spectrometer was calibrated as previously described [77]. The molecular weights were determined through Grams/386 software (Version 3.04, Galactic Industries Corp, Salem, NH, USA) [78] and the m/z value reported indicates the molecular ion, taking into account the most abundant isotope of each element in the molecule.

^1^H-NMR spectra were acquired using a ^UNITY^INOVA instrument (Varian, Agilent Technologies, Santa Clara, CA, USA) operating at 500 MHz (^1^H), setting the sample temperature at 27 °C. Spectra acquisition and processing were performed using VnmrJ software (Version 2.2C, Varian, Agilent Technologies, Santa Clara, CA, USA). The sample was dissolved in deuterated chloroform and the chemical shifts were expressed in ppm (compared to the TMS signal).

Gel permeation chromatography (GPC) experiments were performed using a PL-GPC 110 (Polymer Laboratories, Agilent Technologies Deutschland GmbH, Böblingen, Germany) thermostated system, equipped with two Mixed-D and one Mixed-E PL-gel 5 μm columns joined in series. The instrument is interfaced with a differential refractometer (DR) connected in parallel with a UV-Vis spectrophotometer (Hewlett Packard series 1050, Agilent Technologies, Waldbronn, Germany), and a DAWN multi-angle laser light scattering (Wyatt Technology, Santa Barbara, CA, USA) detector, connected in series. The analyses were performed at 35 ± 0.1 °C using tetrahydrofuran (THF) as an eluent at a flow rate of 1 mL/min. The acquired data were analyzed with ASTRA software (version 6.0.1.10, Wyatt Technology, Santa Barbara, CA, USA) [79].

Thermogravimetric analyses were performed in the range from 50 to 800 °C (thermal ramp of 10 °C min^−1^) and air atmosphere (60 mL min^−1^) with a Perkin-Elmer TGA 7 equipped with a TAC 7/DX (Perkin Elmer, Waltham, MA, USA).

UV-Vis spectra were acquired using quartz cuvettes (1 cm path length) and THF or water as solvents (T = 25.0 ± 0.1 °C) using a Cary60 UV-Vis spectrophotometer (Agilent Technologies, Santa Clara, CA, USA). UV-Vis-diffuse reflectance (UV–Vis DRS) spectra, in the range of 200–800 nm, were recorded on TiO_2_, NC and NCP_A_ as powders and the bandgap energies (E_g_) of the samples were estimated by plotting the modified Kubelka– Munk function [71], using a Jasco V-670 spectrophotometer equipped with a Jasco ISV-722 integrating sphere (Jasco Corporation, Tokyo, Japan).

Fluorescence spectra were acquired with a FP-8200 spectrofluorimeter (Jasco Corporation, Tokyo, Japan), in quartz cells (1 cm path length), using THF as a solvent (T = 25.0 ± 0.1 °C).

The UV-Vis and fluorescence spectra were processed using the Spectragryph optical spectroscopy software (version 1.2.11, Dr. F. Menges, Oberstdorf, Germany, 2019) [80].

## 3. Results and Discussion

To improve the performance of TiO_2_ nanophotocatalyst exploiting only visible light radiation, a suitable organic photosensitizer working as a light-harvesting antenna was chosen. To provide an effective contact between the photosensitizer and the photocatalyst, the nanocomposites were synthesized by a one-pot reaction through a bulk in situ radical co-polymerization of MMA and MAP in the presence of TiO_2_ nanoparticles (Scheme 1, Step 2).

**Figure 2:**
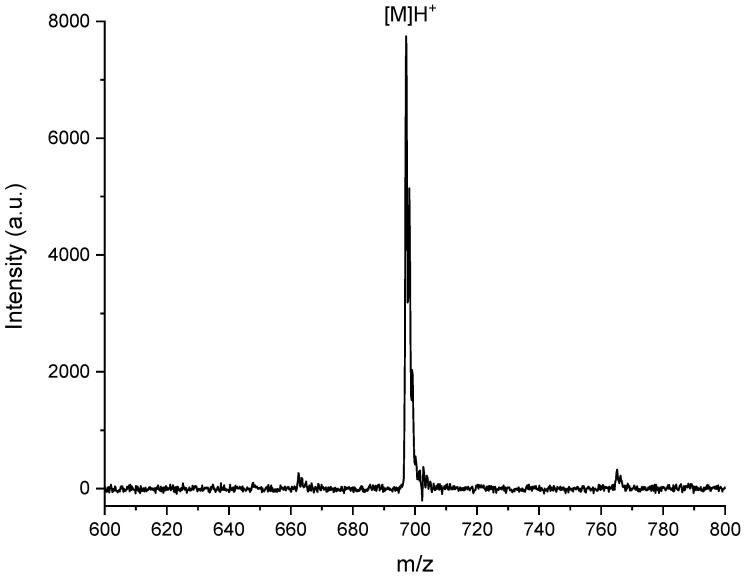
MALDI-TOF mass spectrum of MAP.

In particular, the photosensitizer acrylic monomer MAP was obtained by Steglich amidation of PNH_2_ with methacrylic acid in a CH_2_Cl_2_ anhydrous solution (Scheme 1, Step 1). The chemical structure of MAP was confirmed through MALDI-TOF MS and NMR spectroscopy. The MALDI-TOF mass spectrum of MAP macromonomer (Figure 2) shows a molecular peak at *m/z* = 698.9 Da, attributed to the [M]H^+^ species.

**Figure 3:**
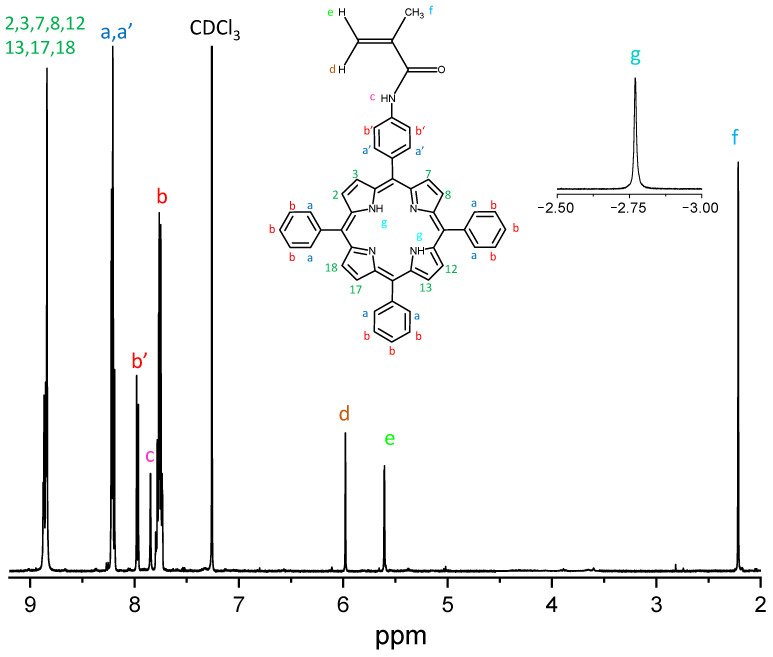
^1^H-NMR spectrum of the MAP macromonomer. In the inset, the singlet of the N-H pyrrole protons, and the MAP structure.

The chemical structure of MAP was confirmed through NMR experiments. The ^1^H-NMR spectrum (CDCl_3_, 500 MHz, Figure 3) shows a multiplet at 8.85 ppm (8H, C-H pyrrole protons in **2,3,7,8,12,13,17,18** positions); two unresolved multiplets at 8.21 ppm (8H, C-H phenyl protons, **a, a’**); a multiplet at 7.96 ppm (2H, C-H phenyl protons, **b’**); a singlet at 7.85 ppm (1H, N-H amide proton, **c**); a multiplet at 7.75 ppm (9H, C-H phenyl protons, **b**); a singlet at 5.97 ppm (1H, C-H vinyl proton, **d**); a singlet at 5.60 ppm (1H, C-H vinyl proton, **e**); a singlet at 2.21 ppm (3H, C-H methyl protons, **f**); a singlet at −2.78 ppm (2H, N-H pyrrole protons, **g**).

The confirmation of those attributions was obtained by ^1^H-^1^H COSY experiment, which shows the correlations: **a’**–**b’**; **a**–**b** (Figure S2).

**Figure 4:**
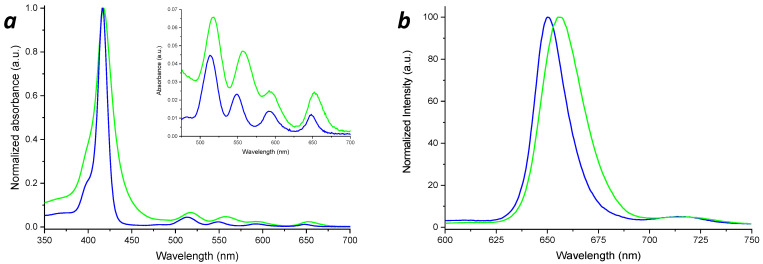
UV-Vis (**a**) and fluorescence (**b**) spectra of the MAP (blue lines) and PNH_2_ (green lines).

The UV-Vis spectrum of PNH_2_ (Figure 4a) shows the Soret band at λ = 418 nm, and the Q-bands at about 517, 557, 592 and 652 nm. According to Gouterman’s four orbitals model [81,82], the Soret band originates from the S_0_-S_2_ electronic transition, while the Q-bands are due to the S_0_-S_1_ electronic transitions [48].

In the case of the MAP spectrum, the Soret band is centered at a λ = 416 nm and the Q-bands at 513, 548, 591 and 648 nm, respectively, revealing a blue shift, with respect to PNH_2_, phenomenon probably due to the amidation of the porphyrin. The MAP molar extinction coefficient in the THF solution was experimentally determined (ε_416nm_ = 381,440 M^−1^ × cm^−1^ ± 2%).

As a confirmation of the porphyrin functionalization, as shown in Figure 4b, due to the influences of the methacrylate group towards the porphyrin HOMO-LUMO orbitals, the fluorescence emission of MAP (λ = 650 nm, λ_exc_ = 416 nm) exhibited a blue shift with respect to PNH_2_ (λ = 656 nm, λ_exc_ = 418 nm).

The photocatalytic nanocomposite was obtained through a bulk in situ radical polymerization reaction (Scheme 1, Step 2). Briefly, MMA and suitable amounts of MAP were mixed with TiO_2_ NPs and sonicated to ensure the adsorption of the sensitizer and MMA onto the photocatalyst. Then, the bulk in situ radical polymerization was initiated through a thermal initiator (AIBN). In this way, three different sensitized photocatalytic nanocomposites, named NCP_A_, NCP_B_ and NCP_C_, each containing a fixed amount of photocatalyst (about 5%w) and an increasing MAP amount (0.5%w, 1.7%w and 7.9%w, respectively) were produced.

**Figure 5:**
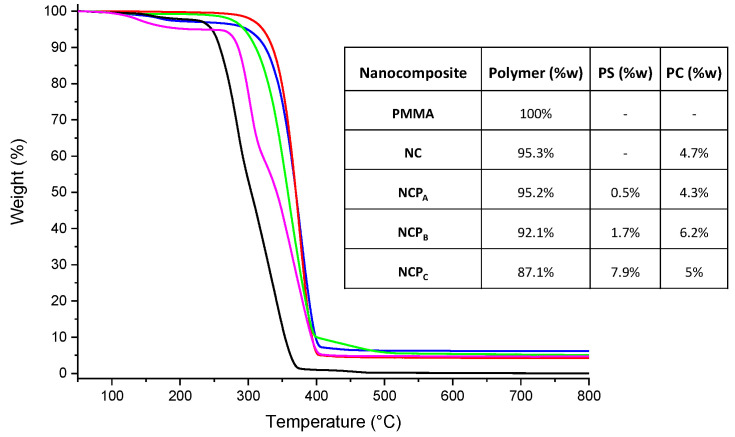
TGA traces of PMMA (black line), NC (magenta line), NCP_A_ (red line), NCP_B_ (blue line) and NCP_C_ (green line). In the inset: the content of polymer matrix, photosensitizer (PS) and photocatalyst (PC) in each nanocomposite.

To determine the TiO_2_ NP content in the nanocomposites, thermogravimetric analyses (Figure 5) were conducted in an air atmosphere (60 mL/min). Because of the TiO_2_ presence, the nanocomposites exhibited a higher degradation temperature than that of the PMMA (T_onset_ = 279 °C). In particular, NCP_A_ (red line), NCP_B_ (blue line) and NCP_C_ (green line) showed a T_onset_ of 346 °C, 338 °C and 317 °C, respectively. The TiO_2_ content of the nanocomposites obtained by TGA revealed that NCP_A,B,C_ contain 4.3%, 6.2% and 5% (w) of TiO_2_ NPs, respectively (see inset in Figure 5).

The amount of MAP in the nanocomposite was determined through UV-Vis spectroscopy by analyzing the supernatant resulting from the nanocomposite purification (see inset in Figure 5). The quantitative determination of the photosensitizer content into the nanocomposites is important to shed light on the photosensitizer influences on the photocatalytic efficiency of the nanocomposite.

**Figure 6:**
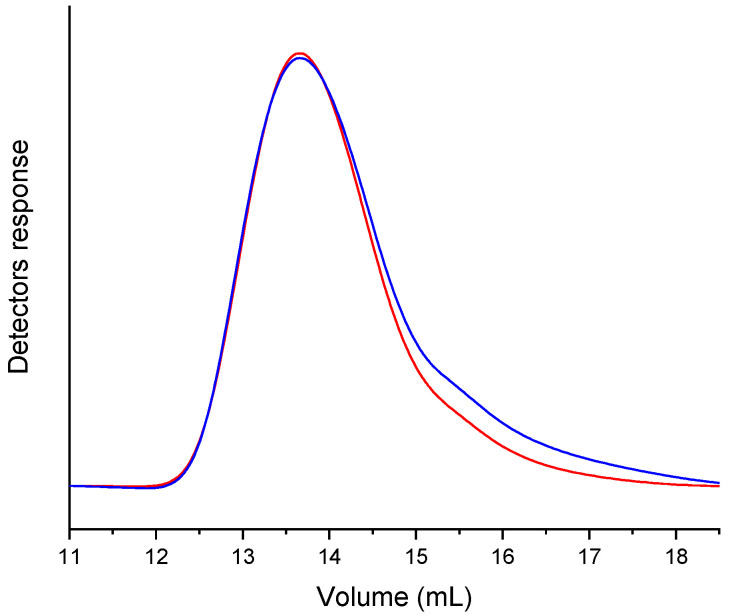
GPC traces of the soluble fraction (in THF) of the nanocomposite NCP_B_, acquired with UV-Vis (set at 416 nm, blue line) and RI (red line) detectors connected in parallel.

To verify the presence of MAP in the polymeric chain, a GPC acquisition was performed using both RI and UV-Vis (set at λ = 416 nm) detectors (Figure 6, NCP_B_ GPC traces). The chromatographic traces showing the overlap of the detectors’ signals confirmed the covalent bond of the photosensitizer within the polymer chains. GPC analyses performed on the other nanocomposites have shown similar results.

To investigate the effects of the in situ approach and the synergistic interactions between photosensitizer and photocatalyst within the nanocomposites, and their correlation with the photodegradation performances, an opposite counterpart physical blend model was developed, by mixing a THF solution of copoly MMA-MAP with TiO_2_ NPs (5%w).

The photocatalytic performances of the sensitized nanocomposites were determined by studying the photodegradation of methylene blue (MB) and rhodamine B (RB) in aqueous solutions, using a solar-radiation-mimicking lamp and using the nanosystems deposed in the form of thin films (see Materials and Methods for details).

Taking advantage of the pseudo-first-order kinetic photodegradation of MB and RB, the degradation kinetics (k) were obtained from the linear fit slope of the ln(C/C_0_) vs. time plot.

**Figure 7:**
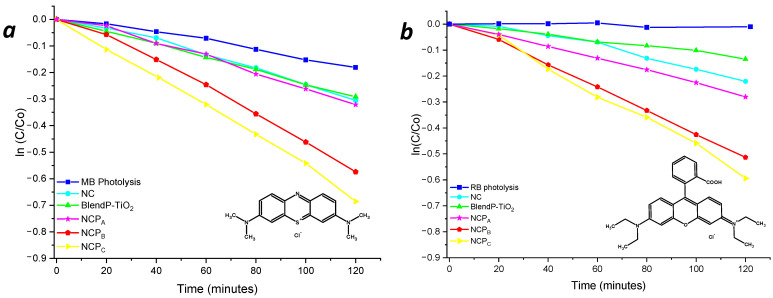
Photodegradation kinetics of MB (**a**) and RB (**b**) in the presence of thin films of nanocomposites and blend.

To highlight the performances of the thin films, considering the active photocatalyst content, the degradation kinetics were also normalized (norm.k) by the weight of the photocatalyst embedded in the nanocomposite. The photolysis phenomenon of the dyes under light radiation was previously verified to avoid any overestimation of the nanocomposites’ performances (Figure 7, blue line).

Despite the comparable content of TiO_2_ NPs inside the NCP nanosystems, the presence of the photosensitizer led to an increase of the photodegradation performances, for both methylene blue and rhodamine B. The comparison of the degradation kinetics obtained with NCP_A_ and BlendP-TiO_2_, having similar content of both photosensitizer and photocatalyst, revealed the higher efficiency of the first than the BlendP-TiO_2_, both in the case of methylene blue (NCP_A_ norm.k = 4.3 min^−1^×g^−1^; BlendP-TiO_2_ norm.k = 3.1 min^−1^×g^−1^) and rhodamine B (NCP_A_ norm.k = 3.5 min^−1^×g^−1^; BlendP-TiO_2_ norm.k = 1.4 min^−1^×g^−1^). This behavior could be attributed to the intimate contact between the photocatalyst and the photosensitizer when the latter and the MMA can penetrate inside the porosities of the TiO_2_ before the polymerization. Instead, for what concerns the physical blend, the copolymeric matrix, due to its high hydrodynamic radius, could not enter inside the TiO_2_ pores.

Nevertheless, the photocatalytic efficiency of NCP_A_ (having low photosensitizer content) was only slightly higher than that of the sensitizer-free nanocomposite NC (see Table 1), which suggests the photosensitizer concentration dependency of the photocatalytic process. Indeed, the photodegradation kinetics of NCP_B_ and NCP_C_ revealed a considerable improvement with respect to NCP_A_ (Figure 7 and Table 1, red and yellow lines, respectively).

**Figure 8:**
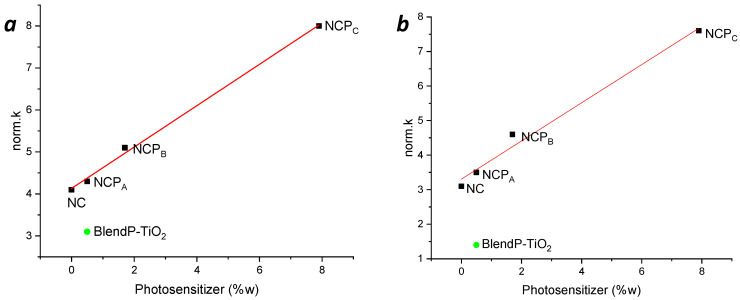
Variation of the norm.k as a function of the nanocomposites’ photosensitizer content (%w) in MB (**a**) and RB (**b**) photodegradation. Green full circles, norm.k of BlendP-TiO_2_.

Hence, to understand the influence of the photosensitizer concentration on the nanocomposites’ photodegradation efficacy, the variation of the norm.k as a function of the photosensitizer content (%w) into the nanocomposites was investigated. In Figure 8, the norm.k in presence of methylene blue (**a**) and rhodamine B (**b**) are reported. It is possible to see a clear linear correlation between the content of the photosensitizer and the photocatalytic performance of the nanocomposites.

**Figure 9:**
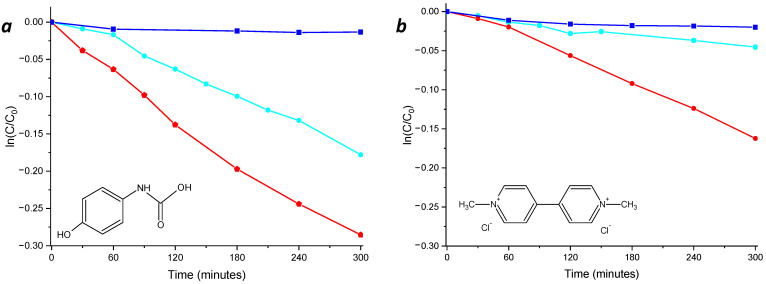
Photodegradation kinetics of acetaminophen (**a**) and paraquat (**b**) in the presence of thin films of NC (cyan line) and NCP_B_ (red line) and their photolysis (blue line).

The efficiency of the nanocomposites was also investigated in the presence of actual wastewater xenobiotic contaminants, such as paraquat (a herbicide) and acetaminophen (a drug). The experiments were performed under light irradiation until 300 min of exposition time and using a solar-mimicking lamp. In particular, the NCP_B_ (Figure 9a,b, red line) was able to induce photodegradation of 25% of acetaminophen and 15% of paraquat, while for the unsensitized NC, only the 16% of acetaminophen and 5% of paraquat was induced, confirming the improved efficiency of the sensitized nanocomposite.

**Figure 10:**
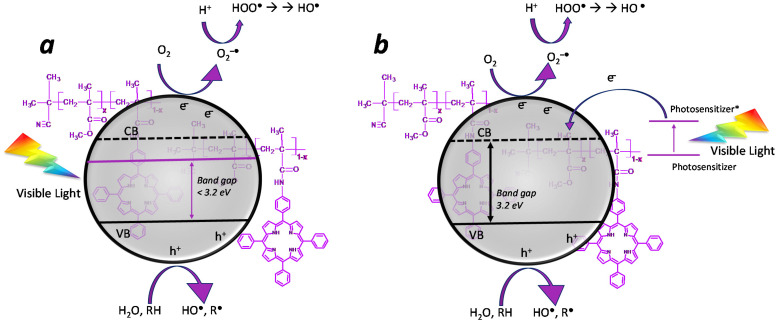
Simplified photocatalytic mechanisms in the presence of a photosensitizer. (**a**) The photosensitizer acts as a dopant, reducing the bandgap. (**b**) The photosensitizer acts as a light-harvesting system by injecting photo-excited electrons into the CB of TiO_2_.

Given that the porphyrinic photosensitizer can act as a light-harvesting antenna, catching the visible range of solar radiation [45], it is necessary to shed light on the mechanisms that enhance photocatalytic performances. In particular, two mechanisms are potentially supposed to take place in presence of the photosensitizer: (i) the photosensitizer acts as a doping system on the semiconductor, decreasing its bandgap and shifting it in the visible range (Figure 10a); (ii) the photosensitizer acts as a light-harvesting system, catching the visible light and generating an electronic promotion from HOMO to LUMO. In this case, if the photosensitizer is near enough to the photocatalyst, the electron transfer process from the LUMO of the photosensitizer to the conduction band of the photocatalyst can take place by direct injection [83] (Figure 10b), avoiding the charge recombination phenomenon (which is a deactivation mechanism of the photocatalyst).

Both phenomena can determine an increase in photo-oxidative performance in the presence of both broad-spectrum solar radiation (with the contribution of both UV and visible light) and source emitting in the visible radiation range (>390 nm).

**Figure 11:**
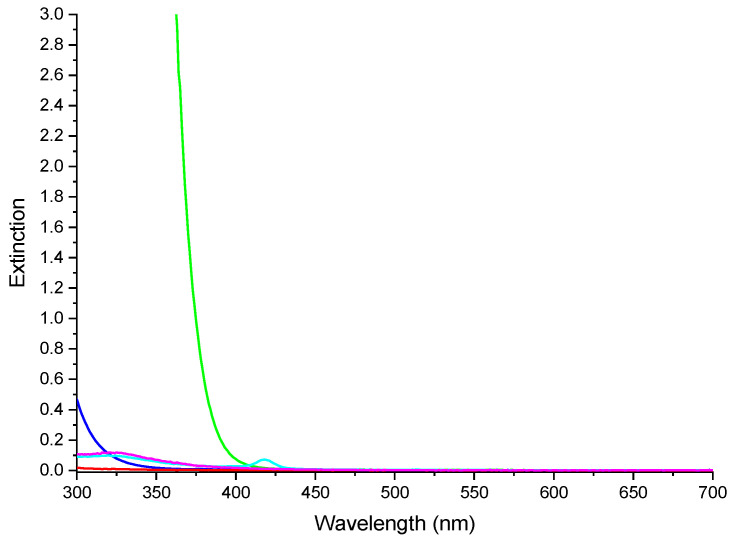
UV-Vis spectra of the reactor glass (blue line), optical filter (green line), PMMA (red line), NC (magenta line) and NCP_B_ (cyan line).

To simulate the artificial illumination systems employed inside places that cannot exploit the direct solar light, such as underground or out-of-Earth orbit structures [26,27], and to investigate the photochemical effects taking place within the photosensitized nanocomposites during photocatalytic processes, photocatalytic experiments were performed shielding UV radiations (see Figure 11) through a filtering solution of 1-methyl-5-(p-toluoyl)-pyrrole-2-acetic acid sodium salt, placed between the lamp and the test tube containing the pollutant solution. In this way, it was possible to evaluate the photocatalytic activity in methylene blue degradation induced by visible light radiation.

**Figure 12:**
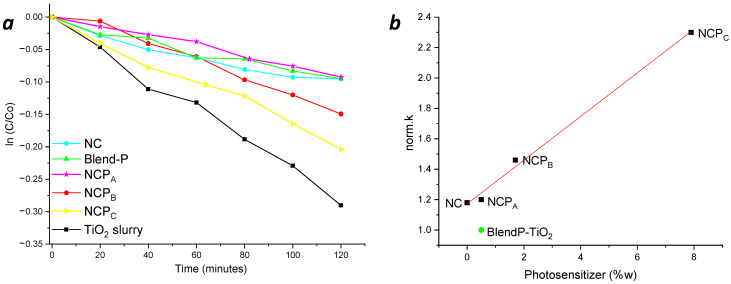
(**a**) Photodegradation kinetics of MB in the presence of thin films of nanocomposites and blend, using only visible range light irradiation. (**b**) Correlation between the norm.k and the photosensitizer content (%w) obtained by the photodegradation of MB (in presence of the chemical filter).

The photocatalytic tests performed using light irradiation in the visible range (Figure 12a) showed that in the presence of either NC, NCP_A_ or BlendP-TiO_2_, the photodegradation kinetics were comparable. However, raising the content (%w) of photosensitizer inside the nanocomposite, as happens for NCP_B_ and NCP_C_, the degradation rate (k) increased (Table 2).

Apparently, a higher photodegradation kinetic was obtained when TiO_2_ in the slurry form was employed as the photocatalyst, probably due to the higher surface area directly exposed to the pollutant solution.

Nevertheless, considering the efficiency of the photocatalyst expressed as norm.k, the NCP_C_ exhibited higher efficiency than that of the TiO_2_ slurry (2.3 and 2.02 min^−1^ g^−1^, respectively). Moreover, as expected, a linear correlation between the photosensitizer content and the kinetics is evident in photodegradation experiments performed with NCP_B_ (Figure 12a, red line) and NCP_C_ (Figure 12a, yellow line), showing the norm.k variations as a function of the content (%w) of photosensitizer inside the nanocomposites (Figure 12b).

To evaluate the possible effect of the porphyrin acting as a doping agent, diffuse reflectance spectra of the sensitized and un-sensitized nanocomposites and TiO_2_ NPs were recorded and used to obtain a Tauc Plot [84]. By applying the Kubelka–Munk transformation on the diffuse reflectance signal, it was possible to approximate it to the absorption coefficient (α) and use the obtained values to plot (α*h*ν)^1/r^ vs. the photon energy (*h*ν), where *r* is an index related to the nature of the transition.

Considering only the indirectly allowed transitions for both TiO_2_ and the nanocomposites (*r* = 2) [71,85], the calculated value of the bandgaps were 3.26 eV for TiO_2_ NPs, 3.24 eV for NC and 2.66 eV for NCP_B_. For TiO_2_ NPs and NC, the values were in accordance with those in the literature [71,86,87,88], while the band gap of the NCP_B_ was 20% lower with respect to both TiO_2_ NPs and NC. Nevertheless, the photosensitizer could also work through light-activated electron injection from photosensitizer LUMO to TiO_2_ conduction band, taking advantage of the photosensitizer absorption band (λ = 416 nm), which falls within the visible radiation range. Thus, it was not possible to quantitatively discriminate which mechanism (band gap variation or electron transfer phenomena) had a major influence when exploiting the visible light range as an energy source.

## 4. Conclusions

Through the in situ polymerization approach, it was possible to fine-tune the content of the photosensitizer inside a nanocomposite by using ad hoc synthesized macromonomers.

The data shown point out the strength of the in situ approach in the production of the photocatalytic nanocomposites containing the photosensitizer, compared to the systems produced using solvent-mediated physical blends. In particular, the in situ approach improves the photocatalytic performance of the photocatalyst, ensuring more intimate contact between the photocatalyst and the photosensitizer, which is essential for the electron transfer process from the photosensitizer to the semiconductor. Moreover, this approach, by exploiting the waveguide property of PMMA, leads to an optimal transport of light inside the porosities of the catalyst.

The measurements carried out demonstrated the usefulness of the photosensitizer inside the nanocomposite in increasing the efficiency of the photocatalyst in the presence of light in the visible range, overcoming the activity problems related to the high band gap value of the TiO_2_. Indeed, the photosensitizer can act as a light-harvesting system, triggering an enhanced photocatalytic mechanism.

The excitation of the electrons probably first takes place in the porphyrinic dye, which has higher light absorption ability and a smaller bandgap [89,90,91]. Then, the excited electrons are injected into the conduction band of the photocatalyst.

This study demonstrated the usefulness of this approach in building up a new kind of co-polymeric photosensitizer, which is suitable to increase up to two times the photocatalytic performances of traditional semiconductors by only adding a small amount of chromophore when used with both UV and visible light radiation. Moreover, these new co-polymeric photosensitizers are also able to broaden the operativity of semiconductors towards visible light regions in a way that could also exploit lights coming from normal indoor illuminations. Finally, this study could pave the way for a new class of co-polymeric photosensitizers, suitable to be used with different photocatalysts in the form of thin films to aid the normal depolluting systems, both in terrestrial indoor applications and underground or low Earth orbit laboratories, or potentially in lunar shelters.

## Figures and Tables

**Table 1:** Composition of nanocomposites and the related photodegradation kinetic constants of MB and RB.

Methylene Blue						
	**Polymer** **(%w)**	**^a^ Sensitizer** **(%w)**	**^b^ TiO_2_** **(%w)**	**k** **(×10^−3^ min^−1^)**	**TiO_2_** **(mg/100 mL)**	**^c^ norm.k** **(min^−1^×g^−1^)**
NC	95.3	/	4.7	2.9	0.72	4.1 ± 0.20
NCP_A_	95.2	0.5	4.3	2.8	0.65	4.3 ± 0.22
NCP_B_	92.1	1.7	6.2	4.9	0.96	5.1 ± 0.26
NCP_C_	87.1	7.9	5	5.6	0.7	8 ± 0.4
BlendP-TiO_2_	94.5	0.5	5	2.5	0.79	3.1 ± 0.16
**Rhodamine B**						
NC	95.3	/	4.7	2.2	0.72	3.1 ± 0.16
NCP_A_	95.2	0.5	4.3	2.3	0.65	3.5 ± 0.18
NCP_B_	92.1	1.7	6.2	4.4	0.96	4.6 ± 0.23
NCP_C_	87.1	7.9	5	5.3	0.7	7.6 ± 0.4
BlendP-TiO_2_	94.5	0.5	5	1.1	0.79	1.4 ± 0.1

^a^ Photosensitizer content %w in the nanocomposite; ^b^ TiO_2_ content %w in the nanocomposite; ^c^ normalized degradation kinetics with respect to TiO_2_ NPs amount.

**Table 2:** Kinetic degradation of MB employing different thin films under visible irradiation.

	Polymer (%w)	^a^ Sensitizer (%w)	^b^ TiO_2_ (%w)	k (×10^−3^ min^−1^)	TiO_2_ (mg/100 mL)	^c^ norm.k (min^−1^×g^−1^)
TiO_2_ slurry	0	/	100	2.35	1.16	2.02 ± 0.1
NC	95.3	0	4.7	0.85	0.72	1.18 ± 0.06
NCP_A_	95.2	0.5	4.3	0.78	0.65	1.2 ± 0.06
NCP_B_	92.1	1.7	6.2	1.41	0.96	1.47 ± 0.07
NCP_C_	87.1	7.9	5	1.61	0.7	2.30 ± 0.1
BlendP-TiO_2_	94.5	0.5	5	0.76	0.79	1 ± 0.05

^a^ Photosensitizer content %w in the nanocomposite; ^b^ TiO_2_ content %w in the nanocomposite; ^c^ normalized degradation kinetics with respect to TiO_2_ NPs amount.

## Data Availability

Not applicable.

## Supplementary Materials

The following supporting information can be downloaded at: https://www.mdpi.com/article/10.3390/nano12060996/s1, Figure S1: UV-Vis spectra of Acetaminophen, Paraquat, Rhodamine B and Methylene Blue; Figure S2: 1H-1H COSY spectrum of the MAP macromonomer (range, 7–9 ppm).

## References

[1] Shi Y., Ma J., Chen Y., Qian Y., Xu B., Chu W., An D. (2022). Recent progress of silver-containing photocatalysts for water disinfection under visible light irradiation: A review. Sci. Total Environ..

[2] Homaeigohar S. (2020). The Nanosized Dye Adsorbents for Water Treatment. Nanomaterials.

[3] Wang Z., Wu A., Colombi Ciacchi L., Wei G. (2018). Recent Advances in Nanoporous Membranes for Water Purification. Nanomaterials.

[4] Kumar M., Borah P., Devi P. (2020). Priority and emerging pollutants in water. Inorganic Pollutants in Water.

[5] Aftab S., Shabir T., Shah A., Nisar J., Shah I., Muhammad H., Shah N.S. (2022). Highly Efficient Visible Light Active Doped ZnO Photocatalysts for the Treatment of Wastewater Contaminated with Dyes and Pathogens of Emerging Concern. Nanomaterials.

[6] Thirsk R., Kuipers A., Mukai C., Williams D. (2009). The space-flight environment: The International Space Station and beyond. Can. Med. Assoc. J..

[7] Ghosh C., Singh V., Grandy J., Pawliszyn J. (2019). Recent advances in breath analysis to track human health by new enrichment technologies. J. Sep. Sci..

[8] Das S., Pal M. (2020). Review—Non-Invasive Monitoring of Human Health by Exhaled Breath Analysis: A Comprehensive Review. J. Electrochem. Soc..

[9] Perrin E., Bacci G., Garrelly L., Canganella F., Bianconi G., Fani R., Mengoni A. (2018). Furnishing spaceship environment: Evaluation of bacterial biofilms on different materials used inside International Space Station. Res. Microbiol..

[10] Straub J.E., Plumlee D.K., Wallace W.T., Alverson J.T., Benoit M.J., Gillispie R.L., Hunter D., Kuo M., Rutz J.A., Hudson E.K. ISS Potable Water Sampling and Chemical Analysis Results for 2016. Proceedings of the 47th International Conference on Environmental Systems.

[11] Limero T., Wallace W., James J.T. Operational validation of the air quality monitor on the International Space Station. Proceedings of the 44th International Conference on Environmental Systems.

[12] NASA Technical Reports Server. https://ntrs.nasa.gov/citations/20030000981.

[13] Bagdigian R.M., Dake J., Gentry G., Gault M. International Space Station Environmental Control and Life Support System Mass and Crewtime Utilization in Comparison to a Long Duration Human Space Exploration Mission. Proceedings of the 45th International Conference on Environmental Systems.

[14] Barta D.J., Wheeler R., Jackson W., Pickering K., Meyer C., Pensinger S., Vega L., Flynn M. An Alternative Water Processor for Long Duration Space Missions. Proceedings of the 40th COSPAR Scientific Assembly.

[15] Trojanowicz M., Bojanowska-Czajka A., Bartosiewicz I., Kulisa K. (2018). Advanced Oxidation/Reduction Processes treatment for aqueous perfluorooctanoate (PFOA) and perfluorooctanesulfonate (PFOS)—A review of recent advances. Chem. Eng. J..

[16] Huling S.G., Pivetz B.E. (2006). In-Situ Chemical Oxidation.

[17] Chen D., Cheng Y., Zhou N., Chen P., Wang Y., Li K., Huo S., Cheng P., Peng P., Zhang R. (2020). Photocatalytic degradation of organic pollutants using TiO_2_-based photocatalysts: A review. J. Clean. Prod..

[18] Cardoso I.M.F., Cardoso R.M.F., da Silva J.C.G.E. (2021). Advanced Oxidation Processes Coupled with Nanomaterials for Water Treatment. Nanomaterials.

[19] Nosaka Y., Nosaka A.Y. (2017). Generation and Detection of Reactive Oxygen Species in Photocatalysis. Chem. Rev..

[20] Bougarrani S., Sharma P.K., Hamilton J.W.J., Singh A., Canle M., El Azzouzi M., Byrne J.A. (2020). Enhanced Photocatalytic Degradation of the Imidazolinone Herbicide Imazapyr upon UV/Vis Irradiation in the Presence of CaxMnOy-TiO_2_ Hetero-Nanostructures: Degradation Pathways and Reaction Intermediates. Nanomaterials.

[21] Chen H., Nanayakkara C.E., Grassian V.H. (2012). Titanium Dioxide Photocatalysis in Atmospheric Chemistry. Chem. Rev..

[22] Guo Q., Zhou C., Ma Z., Yang X. (2019). Fundamentals of TiO_2_ Photocatalysis: Concepts, Mechanisms, and Challenges. Adv. Mater..

[23] Casals M., Gangolells M., Forcada N., Macarulla M., Giretti A. (2014). A breakdown of energy consumption in an underground station. Energy Build..

[24] Casals M., Gangolells M., Forcada N., Macarulla M., Giretti A., Vaccarini M. (2016). SEAM4US: An intelligent energy management system for underground stations. Appl. Energy.

[25] Carillo P., Morrone B., Fusco G.M., De Pascale S., Rouphael Y. (2020). Challenges for a Sustainable Food Production System on Board of the International Space Station: A Technical Review. Agronomy.

[26] Brainard G.C., Barger L.K., Soler R.R., Hanifin J.P. (2016). The development of lighting countermeasures for sleep disruption and circadian misalignment during spaceflight. Curr. Opin. Pulm. Med..

[27] Brainard G.C., Coyle W., Ayers M., Kemp J., Warfield B., Maida J., Bowen C., Bernecker C., Lockley S.W., Hanifin J.P. (2013). Solid-state lighting for the International Space Station: Tests of visual performance and melatonin regulation. Acta Astronaut..

[28] Li R., Li T., Zhou Q. (2020). Impact of Titanium Dioxide (TiO_2_) Modification on Its Application to Pollution Treatment—A Review. Catalysts.

[29] Di Valentin C., Pacchioni G. (2013). Trends in non-metal doping of anatase TiO_2_: B, C, N and F. Catal. Today.

[30] Rehman S., Ullah R., Butt A.M., Gohar N.D. (2009). Strategies of making TiO_2_ and ZnO visible light active. J. Hazard. Mater..

[31] Wang L., Fan H., Bai F. (2020). Porphyrin-based photocatalysts for hydrogen production. MRS Bull..

[32] Li K., Lu X., Zhang Y., Liu K., Huang Y., Liu H. (2020). Bi_3_TaO_7_/Ti_3_C_2_ heterojunctions for enhanced photocatalytic removal of water-borne contaminants. Environ. Res..

[33] Prakash J., Sun S., Swart H.C., Gupta R.K. (2018). Noble metals-TiO_2_ nanocomposites: From fundamental mechanisms to photocatalysis, surface enhanced Raman scattering and antibacterial applications. Appl. Mater. Today.

[34] Basavarajappa P.S., Patil S.B., Ganganagappa N., Reddy K.R., Raghu A.V., Reddy C.V. (2020). Recent progress in metal-doped TiO_2_, non-metal doped/codoped TiO_2_ and TiO_2_ nanostructured hybrids for enhanced photocatalysis. Int. J. Hydrogen Energy.

[35] Fiorenza R., Balsamo S.A., Condorelli M., D’Urso L., Compagnini G., Scirè S. (2021). Solar photocatalytic H_2_ production over CeO_2_-based catalysts: Influence of chemical and structural modifications. Catal. Today.

[36] Li J.-J., Weng B., Cai S.-C., Chen J., Jia H.-P., Xu Y.-J. (2018). Efficient promotion of charge transfer and separation in hydrogenated TiO_2_/WO_3_ with rich surface-oxygen-vacancies for photodecomposition of gaseous toluene. J. Hazard. Mater..

[37] Fiorenza R., Bellardita M., D’Urso L., Compagnini G., Palmisano L., Scirè S. (2016). Au/TiO_2_-CeO_2_ Catalysts for Photocatalytic Water Splitting and VOCs Oxidation Reactions. Catalysts.

[38] Bellardita M., Fiorenza R., D’Urso L., Spitaleri L., Gulino A., Compagnini G., Scirè S., Palmisano L. (2020). Exploring the Photothermo-Catalytic Performance of Brookite TiO_2_-CeO_2_ Composites. Catalysts.

[39] Huang Y., Xu H., Yang H., Lin Y., Liu H., Tong Y. (2018). Efficient Charges Separation Using Advanced BiOI-Based Hollow Spheres Decorated with Palladium and Manganese Dioxide Nanoparticles. ACS Sustain. Chem. Eng..

[40] Murcia J., Ávila-Martínez E., Rojas H., Cubillos J., Ivanova S., Penkova A., Laguna O. (2019). Powder and Nanotubes Titania Modified by Dye Sensitization as Photocatalysts for the Organic Pollutants Elimination. Nanomaterials.

[41] Youssef Z., Colombeau L., Yesmurzayeva N., Baros F., Vanderesse R., Hamieh T., Toufaily J., Frochot C., Roques-Carmes T., Acherar S. (2018). Dye-sensitized nanoparticles for heterogeneous photocatalysis: Cases studies with TiO_2_, ZnO, fullerene and graphene for water purification. Dyes Pigment..

[42] Hashim N., Thakur S., Patang M., Crapulli F., Ray A.K. (2016). Solar degradation of diclofenac using Eosin-Y-activated TiO_2_: Cost estimation, process optimization and parameter interaction study. Environ. Technol..

[43] Jiang G., Geng K., Wu Y., Han Y., Shen X. (2018). High photocatalytic performance of ruthenium complexes sensitizing g-C_3_N_4_/TiO_2_ hybrid in visible light irradiation. Appl. Catal. B Environ..

[44] Atta-Eyison A.A., Anukwah G.D., Zugle R. (2021). Photocatalysis using zinc oxide-zinc phthalocyanine composite for effective mineralization of organic pollutants. Catal. Commun..

[45] Krishnan S., Shriwastav A. (2021). Application of TiO_2_ nanoparticles sensitized with natural chlorophyll pigments as catalyst for visible light photocatalytic degradation of methylene blue. J. Environ. Chem. Eng..

[46] Yu G., Zhu B., Shao L., Zhou J., Saha M.L., Shi B., Zhang Z., Hong T., Li S., Chen X. (2019). Host−guest complexation-mediated codelivery of anticancer drug and photosensitizer for cancer photochemotherapy. Proc. Natl. Acad. Sci. USA.

[47] Park J.M., Lee J.H., Jang W.-D. (2020). Applications of porphyrins in emerging energy conversion technologies. Coord. Chem. Rev..

[48] Villari V., Micali N., Nicosia A., Mineo P. (2021). Water-Soluble Non-Ionic PEGylated Porphyrins: A Versatile Category of Dyes for Basic Science and Applications. Top. Curr. Chem..

[49] Mineo P.G., Abbadessa A., Rescifina A., Mazzaglia A., Nicosia A., Scamporrino A.A. (2018). PEGylate porphyrin-gold nanoparticles conjugates as removable pH-sensor nano-probes for acidic environments. Colloids Surf. A Physicochem. Eng. Asp..

[50] Mineo P.G., Vento F., Abbadessa A., Scamporrino E., Nicosia A. (2019). An optical sensor of acidity in fuels based on a porphyrin derivative. Dyes Pigment..

[51] Villari V., Mineo P., Micali N., Angelini N., Vitalini D., Scamporrino E. (2007). Uncharged water-soluble porphyrin tweezers as a supramolecular sensor for α-amino acids. Nanotechnology.

[52] Gulino A., Lupo F., Condorelli G.G., Mineo P., Fragalà I. (2007). Viable Synthetic Route for a Luminescent Porphyrin Monolayer Covalently Assembled on a Molecularly Engineered Si(100) Surface. Chem. Mater..

[53] Micali N., Mineo P., Vento F., Nicosia A., Villari V. (2019). Supramolecular Structures Formed in Water by Graphene Oxide and Nonionic PEGylated Porphyrin: Interaction Mechanisms and Fluorescence Quenching Effects. J. Phys. Chem. C.

[54] Nicosia A., Abbadessa A., Vento F., Mazzaglia A., Mineo P.G. (2021). Silver Nanoparticles Decorated with PEGylated Porphyrins as Potential Theranostic and Sensing Agents. Materials.

[55] Mazzaglia A., Bondì M.L., Scala A., Zito F., Barbieri G., Crea F., Vianelli G., Mineo P., Fiore T., Pellerito C. (2013). Supramolecular Assemblies Based on Complexes of Nonionic Amphiphilic Cyclodextrins and a meso-Tetra(4-sulfonatophenyl)porphine Tributyltin(IV) Derivative: Potential Nanotherapeutics against Melanoma. Biomacromolecules.

[56] Nicosia A., Vento F., Satriano C., Villari V., Micali N., Cucci L.M., Sanfilippo V., Mineo P.G. (2020). Light-Triggered Polymeric Nanobombs for Targeted Cell Death. ACS Appl. Nano Mater..

[57] Zhou J., Rao L., Yu G., Cook T.R., Chen X., Huang F. (2021). Supramolecular cancer nanotheranostics. Chem. Soc. Rev..

[58] Min K.S., Kumar R.S., Lee J.H., Kim K.S., Lee S.G., Son Y.-A. (2019). Synthesis of new TiO_2_/porphyrin-based composites and photocatalytic studies on methylene blue degradation. Dyes Pigment..

[59] Sułek A., Pucelik B., Kuncewicz J., Dubin G., Dąbrowski J.M. (2019). Sensitization of TiO_2_ by halogenated porphyrin derivatives for visible light biomedical and environmental photocatalysis. Catal. Today.

[60] Lü X.-f., Sun W.-j., Li J., Xu W.-x., Zhang F.-x. (2013). Spectroscopic investigations on the simulated solar light induced photodegradation of 4-nitrophenol by using three novel copper(II) porphyrin–TiO_2_ photocatalysts. Spectrochim. Acta Part A Mol. Biomol. Spectrosc..

[61] Gaeta M., Sanfilippo G., Fraix A., Sortino G., Barcellona M., Conti G.O., Fragalà M.E., Ferrante M., Purrello R., D’Urso A. (2020). Photodegradation of Antibiotics by Noncovalent Porphyrin-Functionalized TiO_2_ in Water for the Bacterial Antibiotic Resistance Risk Management. Int. J. Mol. Sci..

[62] Zhang Y., Zhang G.-L., Wang Y.-T., Ma Z., Yang T.-Y., Zhang T., Zhang Y.-H. (2021). In-situ synthesized porphyrin polymer/TiO_2_ composites as high-performance Z-scheme photocatalysts for CO_2_ conversion. J. Colloid Interface Sci..

[63] Rachel A., Subrahmanyam M., Boule P. (2002). Comparison of photocatalytic efficiencies of TiO_2_ in suspended and immobilised form for the photocatalytic degradation of nitrobenzenesulfonic acids. Appl. Catal. B Environ..

[64] Bettini A. (2014). New underground laboratories: Europe, Asia and the Americas. Phys. Dark Universe.

[65] Ianni A. (2020). Considerations on Underground Laboratories. J. Phys. Conf. Ser..

[66] Carter D.L. Challenges with Operating a Water Recovery System (WRS) in the Microgravity Environment of the International Space Station (ISS). Proceedings of the Annual Meeting of the American Society for Gravitational and Space Research (ASGSR) 2017.

[67] Li Y., Zhao H., Yang M. (2017). TiO_2_ nanoparticles supported on PMMA nanofibers for photocatalytic degradation of methyl orange. J. Colloid Interface Sci..

[68] Klaysri R., Wichaidit S., Piticharoenphun S., Mekasuwandumrong O., Praserthdam P. (2016). Synthesis of TiO_2_ -grafted onto PMMA film via ATRP: Using monomer as a coupling agent and reusability in photocatalytic application. Mater. Res. Bull..

[69] Mirhoseini F., Salabat A. (2015). Ionic liquid based microemulsion method for the fabrication of poly(methyl methacrylate)–TiO_2_ nanocomposite as a highly efficient visible light photocatalyst. RSC Adv..

[70] Cantarella M., Sanz R., Buccheri M.A., Ruffino F., Rappazzo G., Scalese S., Impellizzeri G., Romano L., Privitera V. (2016). Immobilization of nanomaterials in PMMA composites for photocatalytic removal of dyes, phenols and bacteria from water. J. Photochem. Photobiol. A Chem..

[71] Nicosia A., Vento F., Di Mari G.M., D’Urso L., Mineo P.G. (2021). TiO_2_-Based Nanocomposites Thin Film Having Boosted Photocatalytic Activity for Xenobiotics Water Pollution Remediation. Nanomaterials.

[72] Miller L.W., Tejedor-Tejedor M.I., Perez Moya M., Johnson R., Anderson M.A. (2000). Photocatalyst-coated acrylic waveguides for oxidation of organic compounds. Studies Surface Sci. Catal..

[73] Ali U., Karim K.J.B.A., Buang N.A. (2015). A Review of the Properties and Applications of Poly (Methyl Methacrylate) (PMMA). Polym. Rev..

[74] Yan X., Ohno T., Nishijima K., Abe R., Ohtani B. (2006). Is methylene blue an appropriate substrate for a photocatalytic activity test? A study with visible-light responsive titania. Chem. Phys. Lett..

[75] Vitalini D., Mineo P., Scamporrino E. (1999). Effect of combined changes in delayed extraction time and potential gradient on the mass resolution and ion discrimination in the analysis of polydisperse polymers and polymer blends by delayed extraction matrix-assisted laser desorption/ionization time-of-flight mass spectrometry. Rapid Commun. Mass Spectrom..

[76] Mineo P., Vitalini D., Scamporrino E., Bazzano S., Alicata R. (2005). Effect of delay time and grid voltage changes on the average molecular mass of polydisperse polymers and polymeric blends determined by delayed extraction matrix-assisted laser desorption/ionization time-of-flight mass spectrometry. Rapid Commun. Mass Spectrom..

[77] Scamporrino E., Vitalini D., Mineo P. (1996). Synthesis and MALDI-TOF MS Characterization of High Molecular Weight Poly (1,2-dihydroxybenzene phthalates) Obtained by Uncatalyzed Bulk Polymerization of O,O’-Phthalid-3-ylidenecatechol or 4-Methyl-O,O’-phthalid-3-ylidenecatechol. Macromolecules.

[78] Scamporrino E., Maravigna P., Vitalini D., Mineo P. (1998). A new procedure for quantitative correction of matrix-assisted laser desorption/ionization time-of-flight mass spectrometric response. Rapid Commun. Mass Spectrom..

[79] Mineo P.G., Foti C., Vento F., Montesi M., Panseri S., Piperno A., Scala A. (2020). Salinomycin-loaded PLA nanoparticles: Drug quantification by GPC and wave voltammetry and biological studies on osteosarcoma cancer stem cells. Anal. Bioanal. Chem..

[80] Menges F. (2019). Spectragryph—Optical Spectroscopy Software. https://www.effemm2.de/spectragryph/.

[81] Spellane P., Gouterman M., Antipas A., Kim S., Liu Y. (1980). Porphyrins. 40. Electronic spectra and four-orbital energies of free-base, zinc, copper, and palladium tetrakis (perfluorophenyl) porphyrins. Inorg. Chem..

[82] Nicosia A., Vento F., Marletta G., Messina G., Satriano C., Villari V., Micali N., De Martino M., Schotman M., Mineo P. (2021). Porphyrin-Based Supramolecular Flags in the Thermal Gradients’ Wind: What Breaks the Symmetry, How and Why. Nanomaterials.

[83] Chowdhury P., Gomaa H., Ray A.K. (2013). Dye-Sensitized Photocatalyst: A Breakthrough in Green Energy and Environmental Detoxification. Sustainable Nanotechnology and the Environment: Advances and Achievements.

[84] Davis E.A., Mott N.F. (1970). Conduction in non-crystalline systems V. Conductivity, optical absorption and photoconductivity in amorphous semiconductors. Philos. Mag..

[85] Fiorenza R., Sciré S., D’Urso L., Compagnini G., Bellardita M., Palmisano L. (2019). Efficient H_2_ production by photocatalytic water splitting under UV or solar light over variously modified TiO_2_-based catalysts. Int. J. Hydrogen Energy.

[86] Beltrán A., Gracia L., Andrés J. (2006). Density Functional Theory Study of the Brookite Surfaces and Phase Transitions between Natural Titania Polymorphs. J. Phys. Chem. B.

[87] Serpone N. (2006). Is the Band Gap of Pristine TiO_2_ Narrowed by Anion- and Cation-Doping of Titanium Dioxide in Second-Generation Photocatalysts?. J. Phys. Chem. B.

[88] Hanaor D.A.H., Sorrell C.C. (2010). Review of the anatase to rutile phase transformation. J. Mater. Sci..

[89] Zhou W., Cao Z., Jiang S., Huang H., Deng L., Liu Y., Shen P., Zhao B., Tan S., Zhang X. (2012). Porphyrins modified with a low-band-gap chromophore for dye-sensitized solar cells. Org. Electron..

[90] Zhang X., Li Y., Chen Z., Li P., Chen R., Peng X. (2021). Molecular engineering of narrow bandgap porphyrin derivatives for highly efficient photothermal conversion. Dyes Pigment..

[91] Xu Z.J., Gao S.L., Lu X.Q., Li Y.Y., Li Y.M., Wei S.X. (2020). Theoretical analysis of the absorption spectrum, electronic structure, excitation, and intramolecular electron transfer of D-A′-pi-A porphyrin dyes for dye-sensitized solar cells. Phys. Chem. Chem. Phys..

