# Supplementary material for: Photosensitized Thermoplastic Nano-Photocatalysts Active in the Visible Light Range for Potential Applications Inside Extraterrestrial Facilities
Source: Nanomaterials (Basel). 2022 Mar 17;12(6):996. doi: 10.3390/nano12060996 (PMC8948973; doi:10.3390/nano12060996)
Supplement: Supplementary file 1 [file nanomaterials-12-00996-s001.zip › nanomaterials-1633858-supplementary.pdf]

nanomaterials

Supplementary Materials

Photosensitized Thermoplastic Nano-Photocatalysts Active in the Visible Light Range for Potential Applications Inside Extraterrestrial Facilities

Lidia Mezzina [1], Angelo Nicosia [1], Fabiana Vento [1], Guido De Guidi [1] and Placido Giuseppe Mineo [1,2,3,*]

[1] Department of Chemical Sciences and INSTM UdR of Catania, University of Catania, V.le A. Doria 6, I-95125 Catania, Italy; lidia.mezzina@phd.unict.it (L.M.); angelo.nicosia@unict.it (A.N.); fabiana.vento@phd.unict.it (F.V.); guido.deguidi@unict.it (G.D.G.)
[2] Institute for Chemical and Physical Processes, National Research Council (IPCF-CNR), Viale F. Stagno d'Alcontres 37, I-98158 Messina, Italy
[3] Institute of Polymers, Composites and Biomaterials, National Research Council (IPCB-CNR), Via P. Gaifami 18, I-95126 Catania, Italy
* Correspondence: placido.mineo@unict.it

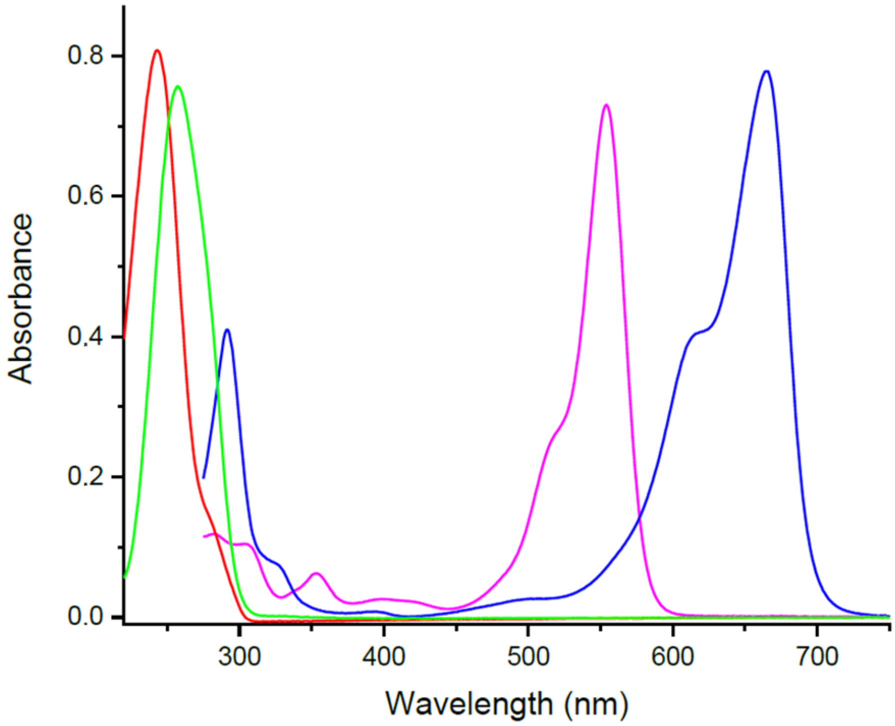

Figure S1. UV-Vis spectra of Acetaminophen (red line), Paraquat (green line), Rhodamine B (magenta line) and Methylene Blue (blue line).

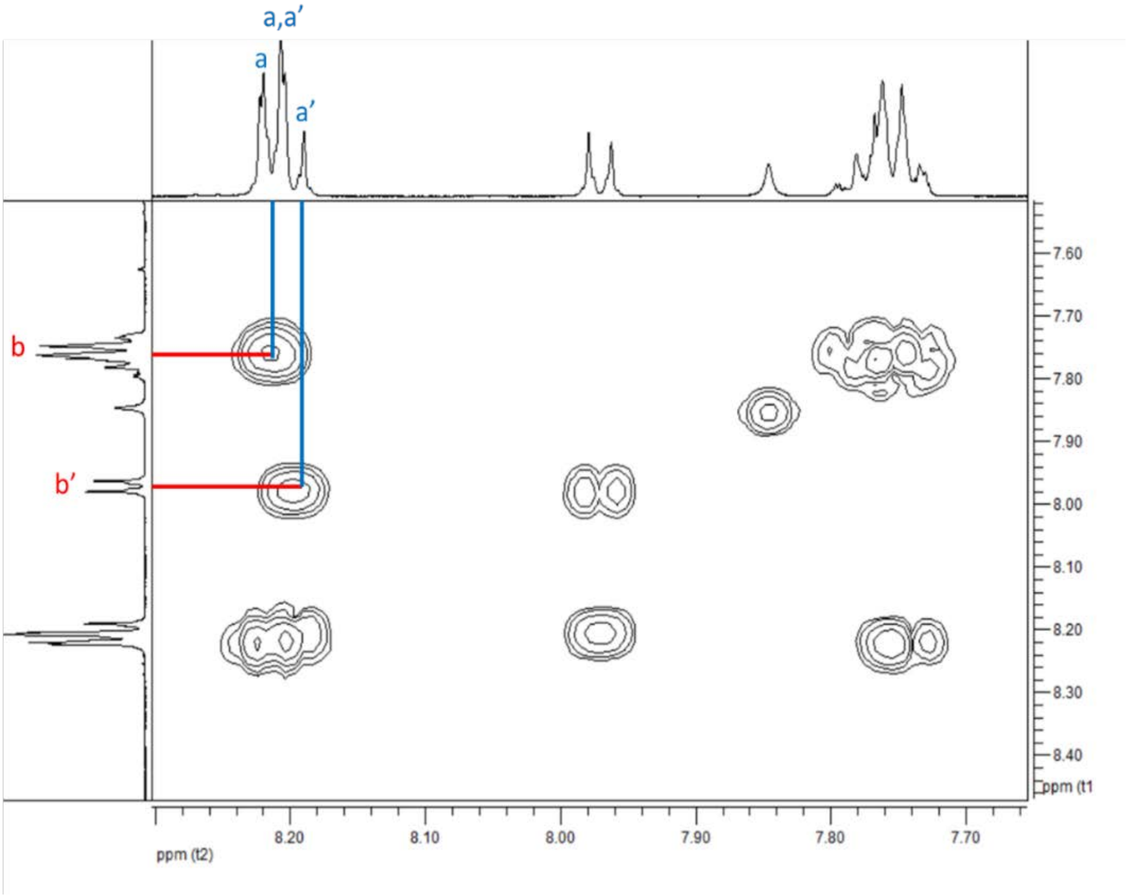

Figure S2. ^{1}H-^{1}H COSY spectrum of the MAP macromonomer (range, 7–9 ppm).
